# *Staphylococcus epidermidis*—key to understanding biofilms, commensalism, and more

**DOI:** 10.1128/jb.00165-25

**Published:** 2025-08-25

**Authors:** Gordon Y. C. Cheung, Michael Otto

**Affiliations:** 1 Pathogen Molecular Genetics Section, Laboratory of Bacteriology, Division of Intramural Research, National Institute of Allergy and Infectious Diseases, U.S. National Institutes of Health 35037, Bethesda, Maryland, USA; Geisel School of Medicine at Dartmouth, Hanover, New Hampshire, USA

**Keywords:** biofilm, commensal immunity, *Staphylococcus epidermidis*, model organism

## Abstract

*Staphylococcus epidermidis*, a ubiquitous inhabitant of human skin, has been known for decades for its capacity to form biofilm-associated infections on indwelling medical devices and has developed into a leading bacterium used in biofilm research. Early investigation on the biofilm matrix focused on *S. epidermidis* “slime,” and currently, *S. epidermidis* represents one of the most prevalent organisms for in-vivo biofilm infection models. More recently, *S. epidermidis* has also become a standard for the investigation of host-commensal interactions on human skin, especially the immune response to colonization that is often called “commensal immunity.” Finally, there are recent efforts to use *S. epidermidis* as a topically applied vehicle for probiotic pathogen exclusion efforts as well as anti-cancer and vaccination strategies.

## INTRODUCTION

**Fig 1:**
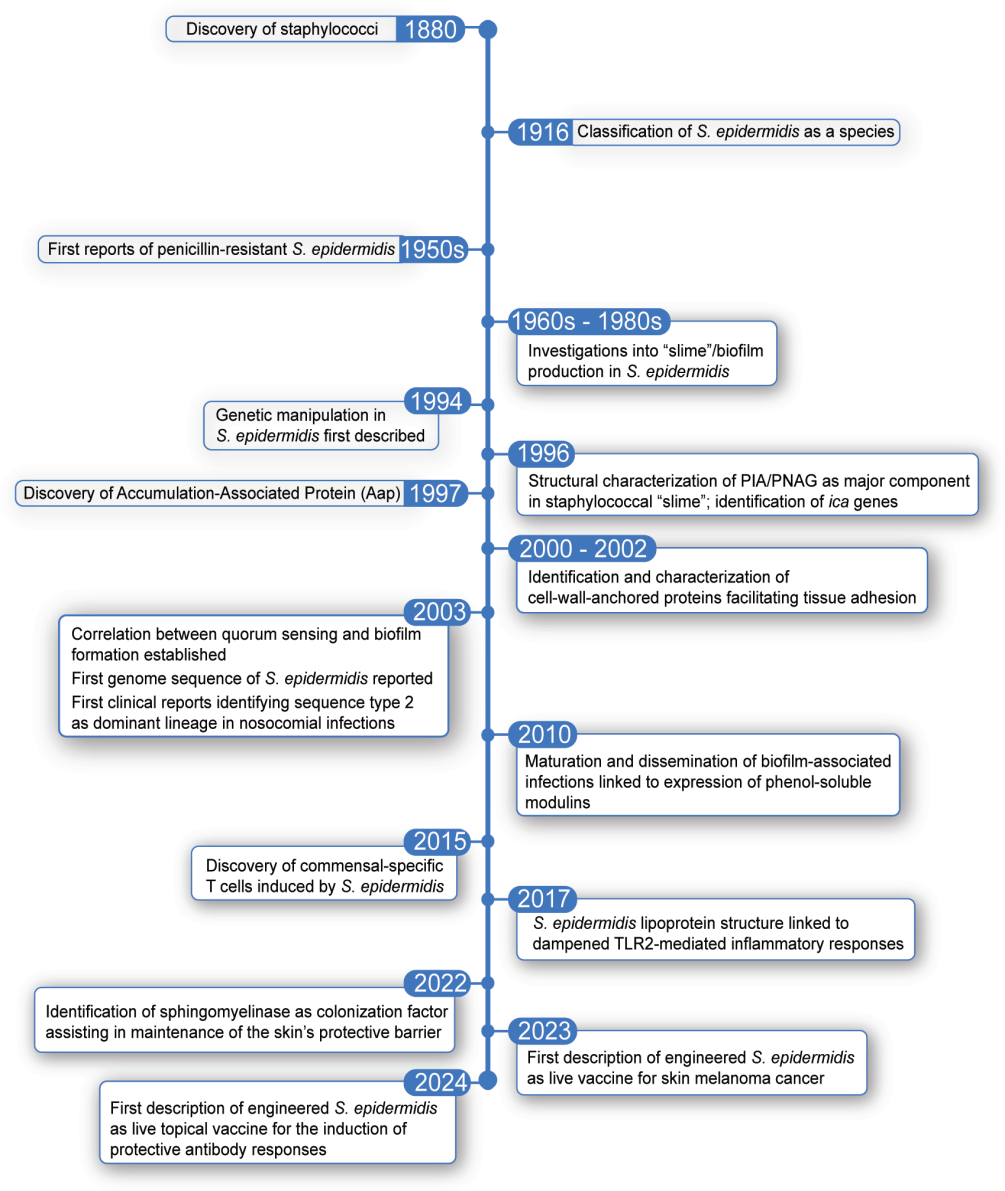
Timeline of major discoveries on *S. epidermidis* physiology, virulence, colonization, and translational use.

The Scottish surgeon Sir Alexander Ogston first described the staphylococci in 1880 (1) (Fig. 1). The term “*Staphylococcus*” is derived from his observation that they characteristically form berry (Greek *kokkos*)-like clusters (Greek *staphylos* for “bunch of grapes”). Four years later, the German physician Friedrich Julius Rosenbach distinguished golden and white staphylococcal colonies and called the golden ones “*Staphylococcus aureus*” (“*aureus*” Latin for golden) and the white ones “*Staphylococcus albus*” (Latin “*albus*” for white) (2). *Staphylococcus albus* was renamed several times in the following decades, and in 1916 finally received the designation we use today, *Staphylococcus epidermidis* (3), with “*epidermidis*” highlighting that it is frequently found on the skin. We now know that there are many species of staphylococci that are white, and even some species other than *S. aureus* that can be yellow or golden, for example, the mouse commensal *S. xylosus* (4). Today’s principal classification of staphylococci rather uses a metabolic phenotype, the ability to coagulate blood, which has coined the term “coagulase-positive staphylococci,” a group that is rather small and comprises *S. aureus*, a major human pathogen that causes a multitude of invasive and non-invasive diseases, while the “coagulase-negative staphylococci” (often abbreviated as CoNS or CNS) are numerous and comprise as one of their most prevalent members *S. epidermidis* (5, 6).

Early reports on *S. epidermidis* (*S. albus*) highlight its predominance not only on the skin but also in the nasal microbiota (7). There were also several reports that have associated *S. albus* with infections, including blood infections and endocarditis (8–11) and even abscesses (12). Based on today’s knowledge, most if not all those reports likely misidentified *S. aureus*, because *S. aureus* can also be white in appearance. This idea is further supported by the fact that those reports characterized the isolates as hemolytic, a phenotype much less pronounced in CoNS. Moreover, abscesses are never caused by *S. epidermidis*.

In the second half of the 20th century, it was increasingly recognized that *S. epidermidis* only rarely is the source of acute, virulent infections, which are more likely caused by *S. aureus*. Rather, it emerged as a leading cause of chronic biofilm-associated infections on indwelling medical devices (13), which had gained interest mostly owing to the increased use of such devices in modern medicine (14, 15). Notably, *S. epidermidis* is also a leading cause of nosocomial blood infections in neonates, elderly patients, and the immunocompromised, mostly because these often originate from biofilm formation on devices (16). The capacity to form biofilms on medical devices and the production of exopolysaccharide, then called “slime,” has been primarily what made *S. epidermidis* a leading pathogen of interest among research on nosocomial infections, biofilms, and the components of the biofilm matrix, a role it still has today (17–19).

**Fig 2:**
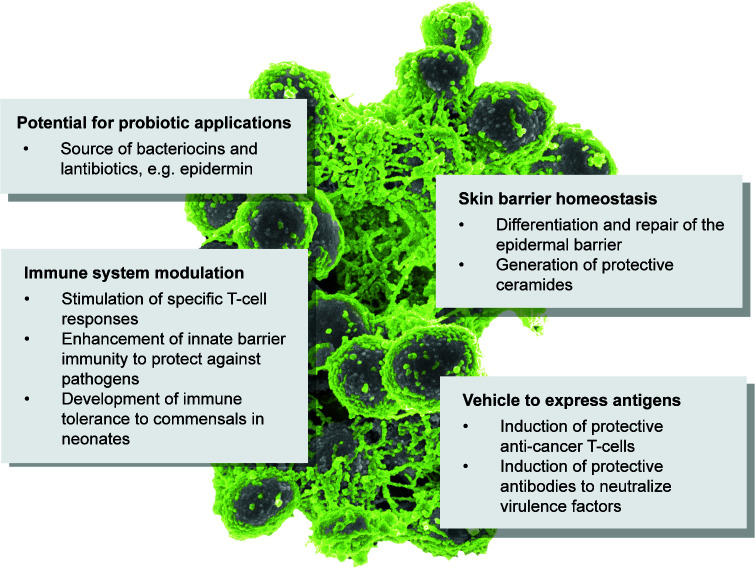
Major areas in which *S. epidermidis* has become a standard of investigation.

While research on *S. epidermidis* until about two decades ago focused on infection and biofilms, the rise of microbiome research that developed because of a revolution in DNA sequencing technology led to the role of *S. epidermidis* as a commensal within the skin microbiome becoming of interest (Fig. 2). That role had, of course, been well known, but it had not been deemed sufficiently interesting as a research subject before. The facts that (i) *S. epidermidis* is a leading constituent of the skin microbiome in sheer numbers and is a ubiquitous skin inhabitant throughout the human population (20, 21) and (ii) genetic tools had already successfully been used owing to research on its pathogenic role have made *S. epidermidis* the standard bacterium used in recent investigation on the mechanisms of skin colonization and the immunology of host-commensal interaction on the skin, and 1 of the 20 most studied bacteria (22). Furthermore, recent research has made use of *S. epidermidis* as a vehicle applied in live form to the skin for potential therapeutic purposes, such as for anti-cancer treatment or as an antigen delivery method for vaccination. Here, we will highlight the three main areas in which *S. epidermidis* has been a standard organism of research: (i) biofilms and biofilm infections, (ii) skin colonization and commensal immunity, and (iii) translational use of a skin commensal as a therapeutic vehicle.

## BIOFILMS AND BIOFILM INFECTIONS

Bill Costerton, the “father of biofilms,” coined the term “biofilms,” which he described as surface-attached agglomerates of bacteria that are enclosed in a matrix of polymeric substances and exhibit different characteristics than free-floating, “planktonic” bacteria (23). Later, the definition of biofilms was adjusted, for example, to also consider non-surface-attached agglomerations as biofilms. Costerton had a strong interest in “medical biofilms,” that is, those forming in the human body by pathogenic bacteria or other microorganisms. This is because biofilm formation considerably increases resistance to all classes of antibiotics and the host’s immune system and represents a huge problem in the clinic, where biofilms can develop, for instance, on indwelling medical devices, such as catheters, prostheses, or implants, or in specific situations such as in the lungs of cystic fibrosis (CF) patients (24). For several decades, the research on medical biofilms focused on the gram-negative pathogen *Pseudomonas aeruginosa*. This pathogen is strongly associated with complications in patients suffering from CF (25), and the focus on *P. aeruginosa* was certainly in part due to the considerable funding *P. aeruginosa* biofilm research received for that reason. Given that there were, and are, no easy animal models to model *P. aeruginosa* CF-associated biofilm infection, research on biofilms—including in Costerton’s “biofilm center” he established at Montana State University—initially was limited strongly to *in vitro* research and “environmental” biofilms, which form for example in wastewater tubing. However, it has been increasingly recognized that many *in vitro* findings in pathogen biofilm research barely reflect the *in vivo* situation during infection.

*S. epidermidis*, together with *S. aureus* and some other CoNS, is the leading pathogen involved with biofilm infections on indwelling medical devices (6, 26–28). Therefore, there would have been more epidemiological reasons to focus on *S. epidermidis* as a standard medical biofilm pathogen from the very beginning of molecular medical biofilm research. However, in contrast to *P. aeruginosa*, it was not considered possible for a long time to genetically manipulate clinical, biofilm-forming *S. epidermidis*. In addition to the funding-related reasons mentioned above, this has certainly contributed to the fact that *S. epidermidis* molecular biofilm research lags that performed on *P. aeruginosa* and still does so as far as *in vitro* research is concerned. However, it is clearly a leading standard biofilm-forming pathogen among the gram-positive bacteria and together with *S. aureus*, a standard for *in vivo* biofilm infection research.

Medical and molecular research on *S. epidermidis* biofilm was spearheaded in Germany. Georg Peters and Gerhard Pulverer in Cologne and then in Munster studied the epidemiology and medical implications of “slime”-producing *S. epidermidis* and its adhesion to medical devices in the 1980s (29, 30). Dietrich Mack in Hamburg described the staphylococcal exopolysaccharide he termed PIA (for polysaccharide intercellular adhesin) as a partially de-acetylated homopolymer of N-acetyl-glucosamine in the 1990s (31). Others have later also called it PNAG (for poly-N-acetylglucosamine) (32). Soon after the structural characterization by Mack, Christine Heilmann and Friedrich Götz in Tübingen discovered the genes that encode production of PIA, calling the locus *ica* (for intercellular adhesin) (33). PIA is not the sole contributor to the biofilm matrix of *S. epidermidis* (the “slime”), and many of the secreted and often cell wall-anchored proteins that also contribute to it were discovered and investigated in Peters’ lab and that of Holger Rohde in Hamburg (34). During that time, Mack’s lab also succeeded in producing transposon mutants in the *ica* locus in biofilm-forming *S. epidermidis* (35), soon followed by heterologous expression of *S. epidermidis ica* genes in *S. carnosus* in Gotz’ lab (36), and finally directed allelic replacement deletion of the *ica* operon in our lab (37). Regulation of the *ica* locus, including via the *icaR* regulator that is encoded next to *the icaADBC* locus, has also gained much attention in the following years (38, 39). These studies established the foundation for in-depth molecular research on *S. epidermidis* biofilms, and most importantly, opened the door to study the impact of specific biofilm genes on biofilm-associated infection in animal models, such as catheter-related infection models. Such models showed that the *ica* locus has a significant contribution to pathogenicity in experimental rodent device-associated infections (40, 41).

Despite the discovery of protein constituents of the staphylococcal biofilm matrix, PIA (PNAG) is still considered the most important contributor to staphylococcal biofilm, and even more so in *S. epidermidis* than *S. aureus* (42). While *S. epidermidis* can form only protein-made biofilms, and strains that only rely on protein biofilms have been isolated from device infections (43), protein-made biofilms appear to be weaker than those formed by PIA (44). Exopolysaccharides produced by bacterial pathogens often differ substantially in structure, and frequently their structure is quite complicated, being made of several different sugars and connections (45). PIA is much simpler in structure, which allows clearer attribution of specific enzymes to its biosynthesis. For example, the enzyme IcaB de-acetylates PIA, which is crucial for its physico-chemical properties, namely its cationic character, retention on the bacterial surface, contribution to *in vivo* pathogenesis, and use as an antigen for vaccination efforts (37, 46). Notably, poly-N-acetylglucosamine is produced by a variety of bacterial biofilm formers (47). In those where genes could be identified, they are homologous to those in the *ica* locus. The research performed on *S. epidermidis* laid the foundation for the considerable volume of future research on bacterial PNAG, including in premier pathogens such as *S. aureus (48*), *Escherichia coli* (49), and *Acinetobacter baumannii* (50).

Among the many surface-located bacterial proteins that have been implicated in staphylococcal biofilms (26, 34), the accumulation-associated protein Aap has special standing as a typical amyloid biofilm protein that has been investigated in depth (51). Aap has been discovered and named early in Georg Peters’ lab (52), while much of the structural and molecular details have later been investigated in the lab of Andrew Herr in Cincinnati. Aap forms amyloid fibrils that protrude from the bacterial surface, potentially connecting staphylococcal cells in agglomerates owing to their high mechanical stability (53). Amyloid formation is dependent on proteolytic trimming of a precursor (54) and the chelation of zinc ions, with zinc driving amyloid fiber formation (55, 56). Aap expression has a significant impact on *in vivo* biofilm-associated infection (57) and represents one of the best-studied biofilm amyloid molecules, an increasing number of which continues to be discovered in a series of biofilm-forming bacteria (58).

*S. epidermidis* also has profound importance for antibiotic resistance research. Not only does it often carry methicillin resistance itself, it also may play a role in transferring that trait to *S. aureus*, giving rise to the notorious methicillin-resistant *S. aureus* (59). However, research on the antibiotic resistance (or strictly speaking, tolerance) that *S. epidermidis* biofilms exhibit on medical devices can be considered even more important, having a general impact on our understanding of biofilm-mediated antibiotic resistance and resistance to host immune defenses. Many studies have investigated the efficacy of specific antibiotics toward biofilms of *S. epidermidis in vitro* and *in vivo* (60), noting strongly increased tolerance values (61). Furthermore, resistance of *S. epidermidis* in biofilms toward phagocytes has been noted early and associated with the biofilm matrix (62). Likely, by covering bacterial cells in a “slime capsule,” the biofilm exopolysaccharide PIA has a role in immune evasion that is not solely linked to its function as a biofilm matrix component (63). The fact that *S. epidermidis* barely stimulates inflammatory responses is likely key to its survival on the skin. One mechanism by which the bacteria achieve this is by modifying cell surface-associated lipoproteins, typically inflammatory in nature, to become less stimulatory for their cognate receptor, Toll-like receptor 2 (64). Generally, the interaction of staphylococcal biofilms with phagocytes and other parts of the host’s immune system has been thoroughly investigated, for example, in the lab of Tammy Kielian, but the focus in that case has been predominantly on *S. aureus* biofilm infections owing to their more aggressive nature (65).

Biofilm research has been closely linked historically with research on quorum-sensing systems. This is because biofilms represent a situation of high cell density. Furthermore, based on early observations in *P. aeruginosa* (66), a notion had developed that quorum-sensing systems are always active in biofilms and therapeutic targeting of quorum-sensing systems is a viable strategy to combat biofilms (67). Research on the staphylococcal quorum-sensing system Agr in *S. aureus* and *S. epidermidis* showed in the early 2000s that this notion does not hold true for other pathogens (68, 69) and was later also adjusted in *P. aeruginosa*, where the relationship is more complicated in part owing to the presence of multiple quorum-sensing systems (70). Namely, it could be shown, by research in our own laboratory, that quorum-sensing (*agr*) mutants of *S. aureus* and *S. epidermidis* form more extended, compact biofilms than the corresponding wild-type strains (68, 69). This behavior was shown to be due mechanistically to Agr-controlled detergent-like peptides called phenol-soluble modulins that function to “loosen up” the biofilm, forming channels and promoting biofilm cluster detachment (44, 71, 72). Furthermore, it was found that staphylococcal *agr* mutants can be frequently isolated and develop *in vivo* during device infection (73–76). This is reminiscent of similar findings also achieved in *P. aeruginosa* (70, 77) and points to a common theme of quorum-sensing and quorum-sensing controlled detachment factors in biofilm maturation, a notion to which research in *S. epidermidis* contributed to a great extent and which has important implications for the potential therapeutic use of quorum-quenching to reduce virulence (78, 79). Further *in vivo* animal research using *S. aureus* and *S. epidermidis* confirmed that these *in vitro* observations also applied to *in vivo* biofilm infection, exemplifying the important role that staphylococcal biofilm research has played in understanding mechanisms of *in vivo* biofilm formation and infection (71, 76).

## BENEFICIAL FUNCTIONS IN THE SKIN MICROBIOME

The first *S. epidermidis* genomes were published in 2003 by Zhang et al., who sequenced the non-biofilm-forming (*ica*-negative) reference strain ATCC12228 (80), and soon later, in 2005, Gill et al., who sequenced the widely used biofilm-forming clinical isolate RP62A (81). During that time, when the number of DNA sequencing studies exploded and there was increased interest in the skin microbiome with *S. epidermidis* as one of its most prominent members, researchers also started to genome-sequence a larger number of *S. epidermidis* strains, focused to a large extent on answering the question whether there is a difference in the genetic composition of strains isolated from the skin versus those isolated from infection. While unequal distribution was found for some selected genes such as *ica*, which had already been reported in earlier studies (82–84), no genes solely associated with infectivity or exclusive commensalism were found, confirming the idea that infection by *S. epidermidis* is an “accident” rather than a survival program of this bacterium (17, 85). While studies investigating the genetic backgrounds of invasive *S. epidermidis* isolates point towards a propensity for particular clonal lineages, such as those belonging to sequence types 2 and 23, as invasive, this is not absolute (86–88). This makes sense considering that infectious strains of *S. epidermidis* do not evolve in an infectious scenario for longer periods of time but represent commensals whose involvement in infection originates from a recent accidental breach of the skin barrier, or contamination during surgery. This notion is also of great importance for the more recent investigation into the beneficial aspects of *S. epidermidis* colonization of human skin and the potential exploitation of *S. epidermidis* for therapeutic purposes. In recent years, *S. epidermidis* has become a standard bacterium that is used in the investigation of host-commensal interaction on the skin, including both the investigation of the immune response to skin commensals and the microbiology of bacterial skin colonization (89). Groundbreaking research in the group of Yasmine Belkaid, who was then at the NIH and is now director of the Institut Pasteur, showed that some strains of *S. epidermidis* belonging to a unique clade induce a specific subset of T cells that enhance innate barrier immunity (90). The fact that in this research, the primary application of an *S. epidermidis* strain limited the proliferation of subsequently applied pathogens has given rise to the idea of a beneficial function of commensal colonization on the skin. Further research into those T cell subsets, from that point on called “commensal-specific T cells,” revealed that they also have a key function in tissue repair (91), they can be stimulated by endogenous retroviruses (92), and they even promote neuron regeneration (93). This line of research has produced fascinating insight into how the response to a skin commensal bacterium can have multiple, and in part beneficial, consequences for the integrity of the skin and far beyond and even integrate viral and bacterial colonization in a multi-kingdom mechanism to control skin homeostasis.

*S. epidermidis* has also been at the center of research on other mechanisms that are beneficial to skin integrity. Elizabeth Grice’s group has shown that a mix of *S. epidermidis* and other skin commensals signals through the keratinocyte aryl hydrocarbon receptor to ensure proper differentiation and repair of the epidermal barrier (94). In our group, we found that virtually all strains of *S. epidermidis* produce a sphingomyelinase that assists the host skin to produce ceramides and thereby skin barrier homeostasis (95). Notably, that enzyme was also crucial for skin colonization of *S. epidermidis* by providing for nutrients and establishing osmotolerance, revealing one of the first factors directly implied in that capacity using deletion mutants and *in vivo* skin colonization models. Finally, *S. epidermidis* was the standard commensal used in research by Tiffany Scharschmidt’s group that revealed that skin colonization during neonatal life is necessary to establish immune tolerance to commensals, based on the influx of activated regulatory T cells into neonatal skin (96). Of special note, that research used a strain of *S. epidermidis* that is a non-biofilm-forming skin isolate and as a special trait produces the lantibiotic epidermin (97). It had not previously been used for purposes other than the investigation of epidermis biosynthesis, even though, in contrast to other *S. epidermidis* strains, it is very easy to transform via electroporation (98). This highlights the recent trend in *S. epidermidis* research that shows a shift from the investigation of pathogenesis and biofilms to commensalism and the concomitant use of different strains, some of which had previously been regarded inappropriate for *in vivo* research on *S. epidermidis* as they are not biofilm-forming.

## A THERAPEUTIC TOOL

There is pronounced recent interest in probiotics, which are live microorganisms used therapeutically. While hitherto investigated mostly for oral use to adjust dysbiosis of the intestinal microbiome and prevent inflammatory and other diseases of the intestinal tract and beyond, skin commensals are now also beginning to be investigated for probiotic use. CoNS have been proposed to control the growth of pathogenic microorganisms based on the production of bacteriocins (99, 100), or more specifically of *S. aureus* via quorum quenching (101). For those purposes, *S. epidermidis* is but one possibility (102, 103), and research so far has been focused on other CoNS (101, 104). This may be in part since in the drug development field, *S. epidermidis* is mostly known as a source of device infections and bacteremia and is thus—despite the planned solely topical application to its natural habitat—not considered a premier choice as a probiotic.

In seminal work that is in part based on Yasmine Belkaid’s discovery of *S. epidermidis*-induced T cell stimulation in the absence of infection or inflammation, Michael Fischbach’s group at Stanford recently used *S. epidermidis* as a vehicle for potential anti-cancer and topical vaccination strategies. His group showed that *S. epidermidis* engineered to express tumor antigens when applied to the skin of mice elicited the production of tumor-specific T cells that promoted mechanisms of cellular immunity at distant sites, where they infiltrated and attacked tumor lesions (105). While the concept of bacteria-mediated cancer therapy is not entirely new (106), the work on *S. epidermidis* stands out regarding the genetic modification procedure and the fact that it includes skin application. An additional advantage of using *S. epidermidis* is that attenuation may not be necessary, which contrasts with other bacteria used in models of anti-tumor applications. In another study by Fischbach’s group, they demonstrated that in addition to the T cell response, *S. epidermidis* elicited an antibody response, predominantly and not surprisingly to the biofilm amyloid protein Aap we discussed above (107). They then showed that *S. epidermidis* engineered to express chimeric proteins of Aap and a conjugated immunogen elicited high antibody titers against that immunogen. This work not only demonstrated that a skin commensal can stimulate a coordinated T and B cell response but offers exciting new avenues for vaccination using topically applied skin commensal bacteria.

## OUTLOOK

*S. epidermidis* has been a leading research object in three categorically different areas: one representing biofilm-associated device infection as a specific type of infection, one that is focused on the immune response to skin colonization, and finally an emerging field where it is proposed as a therapeutic tool. In the first of these three, the microbiology of the organism has been in the center of interest, and biofilm researchers are highly aware that many mechanisms that have been discovered are specific to *S. epidermidis,* and only general principles may be extrapolated to other bacteria. For example, the antibiotic resistance and immunoprotective function of the biofilm matrix is observed across biofilm-forming pathogens, but the molecular difference of biofilm matrix constituents, such as exopolysaccharides and biofilm-associated proteins, remains an extraordinary challenge for the development of anti-biofilm therapeutic strategies. Similarly, while the role of quorum sensing in biofilm development may be more conserved in principle among bacteria than originally thought, the molecular difference of quorum-sensing systems and the involved signals represents a challenge for potential therapeutic intervention even if quorum-quenching is deemed promising for anti-biofilm therapy. In that area, the investigative focus on *S. epidermidis* is thus due more to the frequency at which it causes biofilm infections rather than a genuine “standard” role that would allow extrapolations across different bacteria. While there are occasional reports on anti-biofilm therapeutic strategies claimed to be applicable across different bacterial species and classes, these generally have not passed thorough translational examination, and it is highly likely that anti-biofilm strategies, at least those targeted at the bacteria, will always need to be developed in a pathogen-specific manner. In part for this reason, to this date, the therapy of biofilm infections remains an unmet clinical challenge.

As for *S. epidermidis* being a standard bacterium used in the investigation of commensal immunity, important open questions remain that are related to the underlying microbiology, as most investigations have focused on the immunology of host-commensal interaction on the skin. For example, the molecular microbiology behind the T-cell-stimulatory capacity of *S. epidermidis* remains poorly understood, with the stimulating agents and the involved pathogen recognition receptor(s) remaining undefined. While formylated peptides have been implied (108), no explanation has yet been offered for the strong strain specificity of the effects. Future investigations should address these questions that are of great importance to microbiologists interested in commensal immunity. In that context, it also remains somewhat problematic that a prominent concept of a beneficial function of the skin microbiota has been based predominantly on the response to specific strains that only represent a minority of *S. epidermidis* skin isolates. Therefore, it should also be thoroughly tested whether responses are truly so specific or if the observed specificity potentially arises from the mouse models and experimental setups that were used. This will be crucial to establish if (specific strains of) *S. epidermidis* can continue to represent the standard used to investigate the immunity of host-commensal interaction.

Generally, the investigation of bacterial skin colonization is problematic as it is much more difficult to model in animals than infection, especially when the main interest is in the colonizing bacteria. This is due to several reasons. First, bacteria remain exposed to unwanted mechanical interferences. Second, they must compete with the existing microbiota, or else research must be performed in germ-free mice, which not only drastically increases costs and labor but also creates a highly artificial environment; and removal of the existing microbiota with antiseptics is only transient. Third, mouse skin is very different from human skin, and achieving long-lasting colonization of *S. epidermidis* is extremely difficult. In our laboratory, even very large numbers of applied *S. epidermidis* were found to be very quickly reduced, and only a handful remained within a few weeks, if at all, with pre-existing natural mouse staphylococci, predominantly *S. xylosus*, soon replacing the applied species. Future research should examine whether it makes sense to use mouse commensals such as *S. xylosus* instead of or in addition to *S. epidermidis* in mouse models to investigate the general principles underlying the microbiology and immunology of host-commensal interaction on the skin.

When the purpose of monitoring the effects of engineered *S. epidermidis* in mice is for ultimate probiotic or other use in humans, *S. epidermidis* can and should not be replaced, because its capacity to colonize many areas of human skin throughout the population is what makes it a preferred standard in those cases. Improvements in that area should include developing *S. epidermidis* vehicle strains that can compete with naturally occurring human *S. epidermidis* for successful colonization, which is a prerequisite to obtain the desired effects. This may be achieved, for example, by expressing bacteriocins as weapons of bacterial competition or using strains in which such bacteriocins naturally occur, like the epidermin producer mentioned above.

In conclusion, we believe *S. epidermidis* has rightfully developed a standing as a standard species of use in the area of bacterial biofilms of medical importance and as a bioengineered tool in modern anti-cancer and vaccination strategies using topical skin application. While groundbreaking discoveries have been made by almost exclusively using specific *S. epidermidis* in the investigation of commensal immunity, we believe that the field would benefit from further analyzing microbiological aspects of host-commensal interaction to confirm the applicability of such use.
